# Cerebrovascular CTA radiomics for objective collateral grading in acute ischemic stroke

**DOI:** 10.1186/s41747-026-00680-8

**Published:** 2026-03-16

**Authors:** Dimitrios Rallios, Adam Hilbert, Charles Majoie, Wim Van H. van Zwam, Aad van der Lugt, Martin Bendszus, Susanne Bonekamp, Peter Vajkoczy, Orhun U. Aydin, Dietmar Frey

**Affiliations:** 1 CLAIM – Charité Lab for Artificial Intelligence in Medicine, Charité – Universitätsmedizin Berlin, Berlin, Germany; 2 Department of Neurosurgery, Charité – Universitätsmedizin Berlin, Berlin, Germany; 3 Department of Radiology and Nuclear Medicine, Amsterdam UMC, Amsterdam, Netherlands; 4 Department of Radiology and Nuclear Medicine, Maastricht University Medical Centre, Maastricht, Netherlands; 5 Department of Radiology and Nuclear Medicine, Erasmus MC, Rotterdam, Netherlands; 6 Department of Neuroradiology, Universitätsklinikum Heidelberg, Heidelberg, Germany

**Keywords:** Cerebral angiography, Collateral circulation, Computed tomography angiography, Machine learning, Radiomics

## Abstract

**Objective:**

Collateral circulation is a key determinant of functional outcome after large vessel occlusion (LVO) and informs thrombectomy decisions. However, collateral grading is rater-dependent and error-prone. We developed an automated cerebrovascular radiomics pipeline to establish objective collateral scoring on computed tomography angiography (CTA).

**Materials and methods:**

We retrospectively analyzed admission CTAs from 343 LVO patients in the MR CLEAN trial, split into training/validation (*n* = 274) and testing (*n* = 69) sets. Vessel regions of interest were segmented using nnU-Net models trained on 40 arterial tree CTAs and 125 multiclass circle of Willis (CoW) cases. Radiomics features were extracted from vascular regions. Predictive features were identified, and a random forest classifier was trained to distinguish sufficient (> 50%) from insufficient (≤ 50%) collateral status according to the Tan score system. Performance was compared to the atlas-based middle cerebral artery (MCA) mask model and validated on an external cohort of 140 acute LVO patients.

**Results:**

Segmentation models accurately annotated cerebral arteries (95th percentile Hausdorff distance 4.49, Dice similarity coefficient 0.83) and CoW segments (2.27 and 0.81, respectively). After feature selection, 6 top features were identified for vessel-tree radiomics, 98 for MCA mask-based radiomics, and 32 for a combined vessel-tree/CoW model. Vessel-tree outperformed MCA mask model on both internal (area under the receiver operating characteristic curve (AUROC): 0.88 *versus* 0.82) and external (AUROC: 0.83 *versus* 0.66) test sets. Adding CoW features further improved performance, achieving 0.87 AUROC.

**Conclusion:**

We presented a fully automated generalizable CTA radiomics approach for objective collateral scoring in acute LVO.

**Relevance statement:**

This study introduces a fully automated CTA cerebrovascular radiomics pipeline that objectively assesses collateral status in patients with acute ischemic stroke. Combining vessel-tree and circle of Willis features improved collateral score prediction accuracy and generalizability, supporting more reliable, data-driven decision-making in acute large vessel occlusion management.

**Key Points:**

- Collateral circulation status informs prognosis and guides treatment in acute stroke, but grading is rater-dependent; our pipeline standardizes collateral assessment.
- We propose a CTA radiomics approach, trained and validated on multicenter data, externally tested on an independent cohort, demonstrating high effectiveness and generalizability.
- Automated and reliable collateral scoring has the potential to reduce inter-rater variability, improve workflow efficiency, and support individualized treatment decisions.

**Graphical Abstract:**

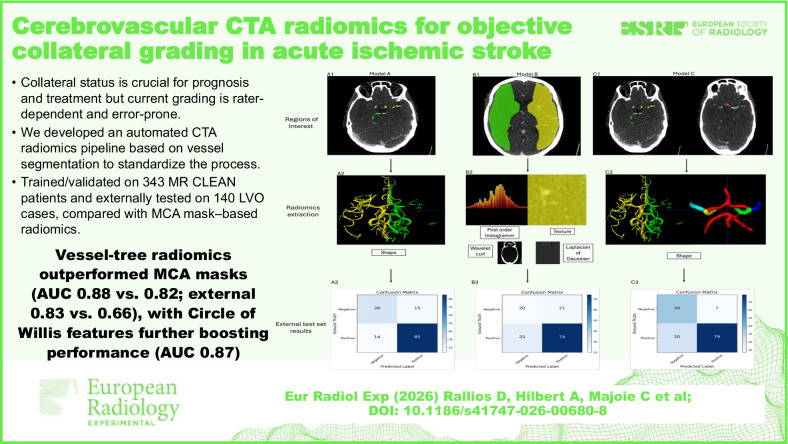

## Background

Acute large vessel occlusion (LVO) is a severe ischemic stroke subtype associated with poor clinical outcomes [1]. Beyond rapid diagnosis, clinical and radiographic assessments are essential to guide individualized treatment decisions [2]. One key imaging parameter is the collateral status—the extent and pattern of contrast opacification in vessels distal to an arterial occlusion [3]. Despite an occlusion, patients with robust collateral circulation can maintain cerebral perfusion via alternative pathways, which helps limit infarct size and improves outcomes [4]. Beyond its role as a prognostic factor, collateral status now emerges as a decision-making criterion, with recent studies highlighting its importance in selecting patients for endovascular thrombectomy [5, 6].

Given the clinical importance of collateral circulation in stroke management, reliable assessment at presentation is essential. CT angiography (CTA), the primary imaging modality for evaluating cerebral vessels in the acute setting, is routinely used to assess collateral status [7]. However, despite efforts toward automation and quantification [8, 9], the assessment remains subjective and variable even when performed by experienced reviewers [10].

Vessel segmentation enables detailed visualization of the cerebral vasculature and has shown value across pathologies, including stroke [11]. However, how well vessel segmentation contributes to supporting clinical decision-making remains underexplored. One way to bridge this gap is through radiomics, which leverages segmented vessels as regions of interest to extract quantitative, image-derived features [12]. This technique has shown promise in stroke diagnosis and outcome prediction by analyzing image-derived patterns and textures [13]. When combined with machine learning, radiomics can further enhance classification performance in clinical tasks such as outcome prediction [13].

In this study, we propose an automated pipeline for predicting collateral status from CT angiography. Vascular structures are segmented using nn-UNet models, and radiomics features are extracted from the cerebral arterial tree and circle of Willis (CoW). A feature selection pipeline identifies the most predictive features, which are used to train a random forest classifier. We compare vessel-based regions of interest to traditional middle cerebral artery (MCA) territory masks on internal and external test sets and assess the added value of incorporating CoW radiomics.

## Materials and methods

### Imaging data

We retrospectively analyzed data from 502 patients enrolled in the MR CLEAN trial, a multicenter randomized study of intra-arterial therapy *versus* standard intravenous thrombolysis for acute ischemic stroke due to proximal anterior circulation occlusion [7]. Our selection process for the inclusion of patients adhered to the following criteria.

**Fig. 1:**
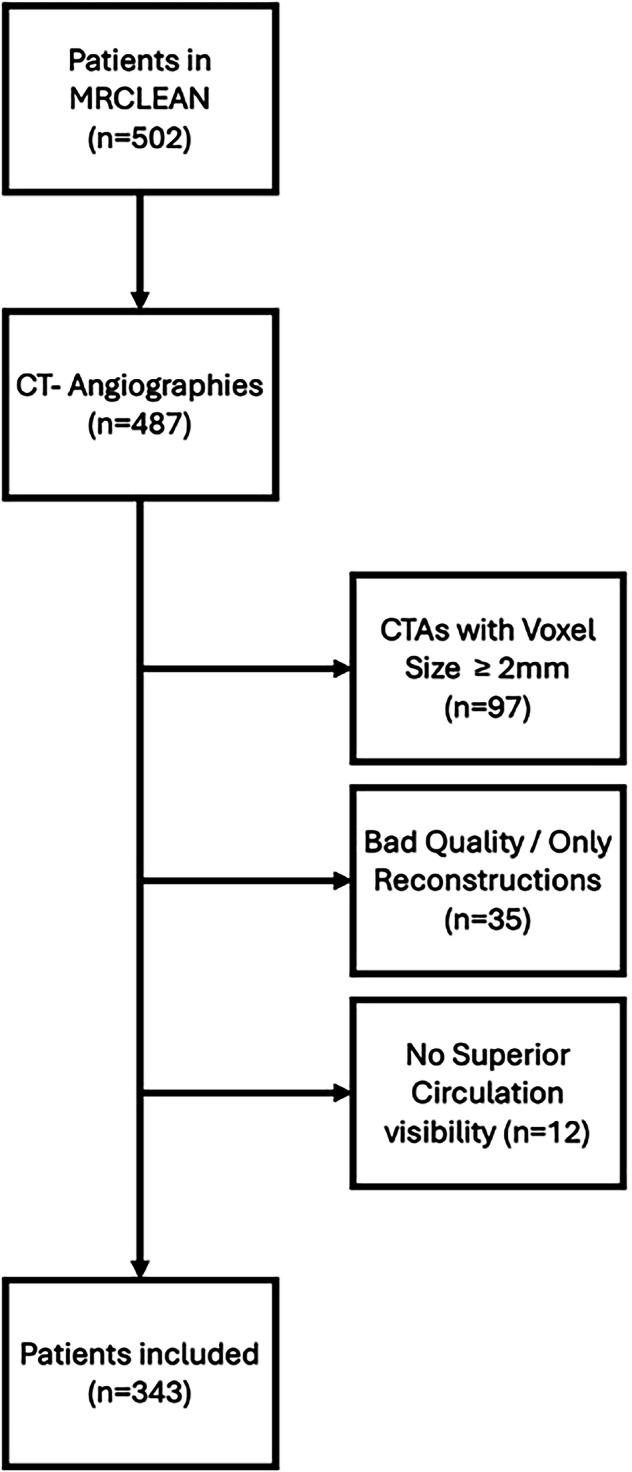
Flow diagram of inclusion from the MR CLEAN study. CTA Computed tomography angiography

We included 343 patients with acute proximal anterior circulation LVO and CTA scans covering the whole brain or at least the lateral ventricles to ensure distal circulation visibility (Fig. 1). To standardize image quality, only scans with slice thickness < 2.0 mm were selected. CTAs were visually assessed, and only patients whose scans were acquired with no motion artifacts were included. Examples of excluded cases are shown in Supplementary Fig. S1. Images were converted to the Neuroimaging Informatics Technology Initiative—NIfTI 1 [14] or processing. This dataset was used for developing the binary cerebral artery tree segmentation model and for training the downstream radiomics-based classifier model.

As an external test set, we included 140 LVO patients from the University of Heidelberg (2018–2021) with admission CTA scans meeting our imaging criteria. All available cases with Tan scores [15] 0–1 (*n* = 41) were included, and additional cases scored Tan 2–3 were randomly selected to match the MR CLEAN distribution.

For CoW segmentation, we used 125 CTA scans from the 2024 TopCoW Challenge dataset [16], which includes annotated stroke-related cases from the University Hospital Zurich (2018–2019). Segmentations were manually reviewed by expert neuroradiologists, neurologists, and neurosurgeons. All scans met our voxel size and image quality criteria.

### Image preprocessing

Brain extraction was performed using SynthStrip (FreeSurfer) to retain only brain slices above the foramen magnum [17]. To standardize imaging across datasets, we registered all CTA scans and segmentation masks to a CT template using rigid transformation with the Linear Image Registration Tool‒FLIRT from the Oxford Centre for Functional Magnetic Resonance Imaging of the Brain‒FMRIB Software Library [18–20] (Supplementary Fig. S2).

### Vessel segmentations

We developed two nn-Unet-based [21] vessel segmentation models: model 1, a binary model for the full vascular tree, and model 2, a multiclass model for CoW segments. Both were trained for 1,000 epochs using default hyperparameters, CT normalization, and five-fold cross-validation.

The binary model was trained using a three-step iterative process. Initial manual segmentations were performed on 10 CTA scans by a junior rater (D.R., medical doctor, 2 years of experience for vessel segmentation), then reviewed and refined by two senior raters (O.U.A., medical doctor, 7 years of experience for vessel segmentation, and D.F., neurosurgeon, 18+ years clinical experience). The refined dataset (*n* = 20) was used to train a second nnU-Net model, which then autosegmented another 20 patients. Expert review and correction produced a final training set of 40 semiautomated segmentations. Final vessel masks were generated by combining the cross-validation models according to their best performance metrics and were subsequently split into left and right hemispheres.

For model 2, we used 125 CTA scans with expert annotations from the TopCoW Challenge 2024 [16]. Vessels were labeled by segment: anterior cerebral artery (including A3), posterior cerebral artery, internal carotid artery, middle cerebral artery (MCA), anterior communicating artery, posterior communicating artery, and basilar artery. For model training, we retained distinct labels for the MCA and internal carotid artery, and merged the remaining vessels into a single class, resulting in five total labels. Both models were trained using a 5-fold cross-validation and were assessed visually by the senior raters and via Dice similarity coefficient and average 95th percentile Hausdorff distance [22].

**Fig. 2:**
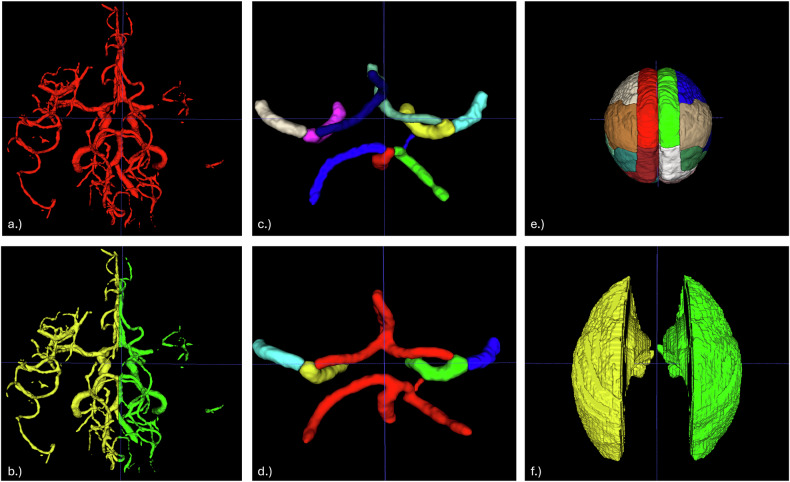
**a** Binary segmentation of the whole cerebral vasculature. **b** Two-labeled segmentation divided for right and left sides. **c** Circle of Willis (CoW) segmentation with individual labeling for each branch. **d** Modified CoW segmentation. **e** Brain atlas showing color-coded labels for regions supplied by each branch of the major arteries. **f** Modified middle cerebral artery atlas

We compared our vessel-based method against MCA arterial territory masks derived from vascular lesion distribution [23]. The MCA mask was chosen because it is widely used in stroke imaging studies [13] and has been applied in automated collateral assessment [9]. As the Tan collateral score evaluates pial filling distal to the occlusion relative to the contralateral side, this mask approximates the region that neuroradiologists visually assess during scoring. To ensure anatomical specificity, the atlas was modified by excluding non-MCA territories and merging all MCA-related subregions into a single composite mask representing the full MCA perfusion territory. We modified the atlas by excluding non-MCA territories and merging all MCA-related regions. A visual summary of segmentation types and modifications is shown in Fig. 2.

### Collateral scoring

Collateral status in both the MR CLEAN trial and the Heidelberg dataset was assessed using the 4-point Tan collateral score [15], which evaluates pial arterial filling distal to an occlusion (0: none, 1: > 0% but ≤ 50%, 2: > 50% but less than 100%, 3: 100%). For modeling, scores were dichotomized into sufficient (2–3) and non-sufficient (0–1).

In MR CLEAN, two experienced neuroradiologists independently graded collaterals according to the Tan scoring system, with a third resolving disagreements. Raters were blinded to clinical data, except for the symptomatic side, and CTA acquisition protocols varied among centers [24]. In the Heidelberg dataset, a single neuroradiologist at the University of Heidelberg performed the grading, either before treatment during clinical routine or retrospectively, without being actively blinded to the clinical outcome but aware of the symptomatic side. CTA was acquired using 75 mL of Accupaque 350 (Siemens Somatom, Siemens Healthineers AG) (4.0 mL/s) bolus triggered.

### Radiomics

We used the PyRadiomics library [12] to extract shape, first-order, and texture features from vessel segmentations, and first-order and texture features from MCA territory masks. Voxel intensities were clipped to 1–500 HU and z-score normalized per scan. Texture enhancement was performed using “coif-1” wavelet and Laplacian of Gaussian‒LoG filters. This configuration yielded 1,094 radiomic features per region of interest (ROI), with an additional 14 shape features extracted for vessel-based ROIs.

**Fig. 3:**
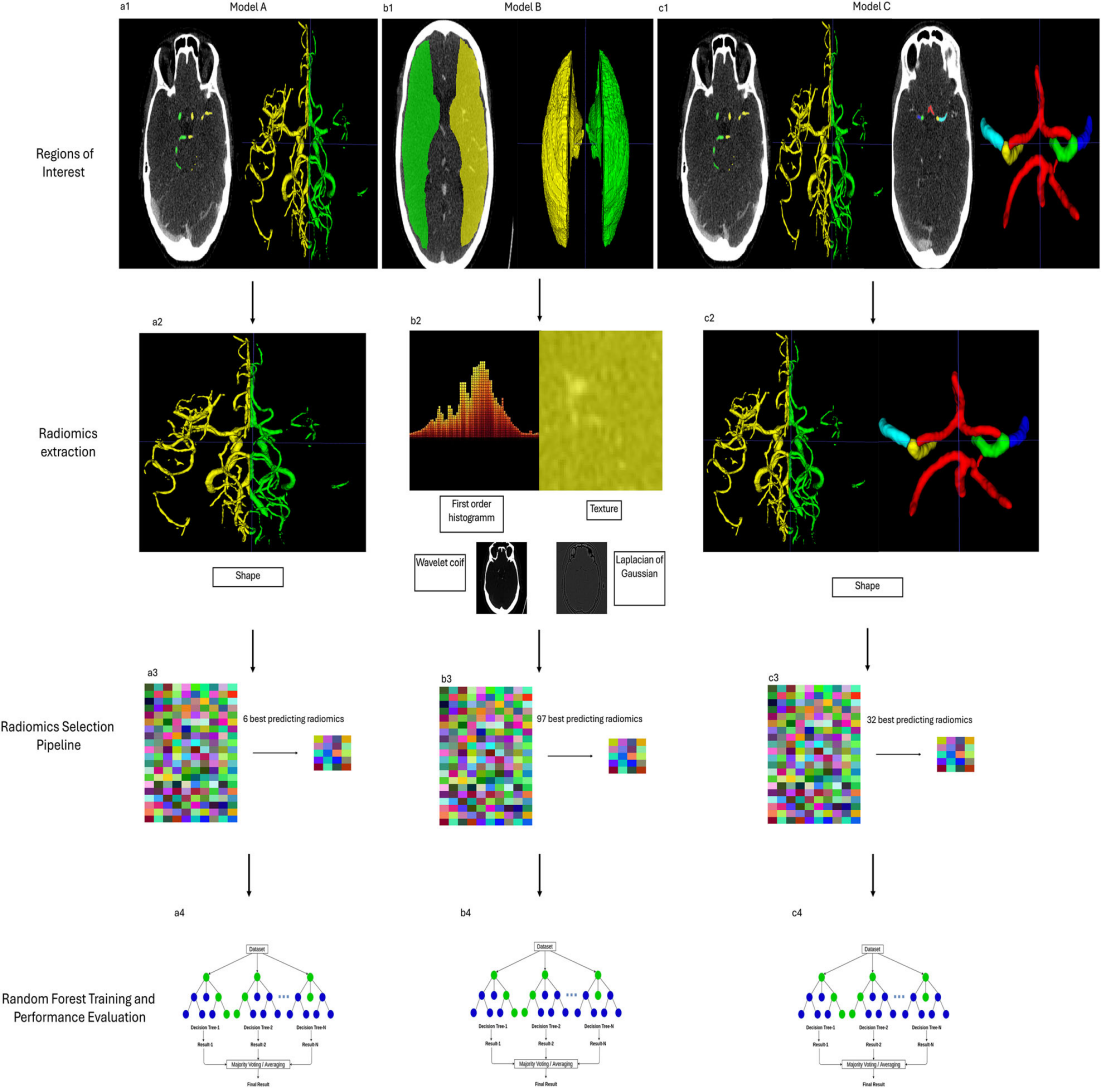
Experimental structure. **a1** Vessel segmentation via model 1. **a2** Shape radiomics extraction. **a3** Identification of best predictive features. **a4** Based on these features, an RFC is trained. **b1** MCA masks are registered onto the CTA image. **b2** Radiomics extraction from original and transformed images. **b3** Identification of best predictive features. **b4** RFC training based on the best radiomics. **c1** Model 1 and model 2 segmentations. **c2** Shape radiomics features for seven different regions of interest (left hemisphere, right hemisphere, left and right ICA, left and right MCA and the communicating arteries, including posterior cerebral artery and anterior cerebral artery) were extracted. **c3** Identification of best predictive features. **c4** RFC training. CTA, Computed tomography angiography; ICA, Internal carotid artery; MCA, Middle carotid artery; RFC, Random forest classifier

An exploratory analysis was conducted on vessel-based ROIs to evaluate three feature sets: (1) texture only; (2) shape only; and (3) combined—used to train model A. For MCA-based ROIs, all extracted features from left and right masks were included in the selection pipeline, forming model B. The best-performing features were then combined with CoW radiomics to construct model C. The experimental design is shown in Fig. 3.

Following feature extraction, all radiomics were scaled to a 0–1 range using min-max normalization. To prevent data leakage, normalization parameters were computed from the training set only and then applied to both internal and external test sets.

### Machine learning protocol

The machine learning protocol of this study was designed to comply with the CLAIM [25] checklist for AI in medical imaging and the CheckList for EvaluAtion of Radiomics research (CLEAR) [26].

### Feature selection process

Since the selection was based solely on the training set (*n* = 274), including all radiomics features for classification would create a high-dimensional feature space, potentially increasing the risk of overfitting and compromising model performance [27, 28]. To address this challenge, we removed highly correlated radiomics features while retaining the most predictive ones. Feature importance was determined via L2-regularized logistic regression optimized for area under the receiver operating characteristic curve (AUROC) using 10-fold stratified cross-validation. From each feature pair with correlation > 0.9, the less important feature was discarded.

To identify the optimal number of features, we iteratively evaluated subsets of top-ranked radiomics using L2-regularized logistic regression with stratified 10-fold cross-validation. Starting with the top 5 features, we selected the subset yielding the highest average Receiver Operating Characteristic (ROC)-AUROC. To enhance model interpretability, SHapley Additive exPlanations (SHAP) [29] plots were generated to depict each model’s best-performing radiomics identified in this step.

Consequently, we trained a random forest classifier to predict collateral status using the most predictive radiomics features. Hyperparameters were tuned via Bayesian optimization (Optuna [30]), over 50 trials, maximizing average AUROC [31]. The final model (1,000 trees) was validated with 4-fold cross-validation (Supplementary Fig. S3). For preprocessing, feature selection, and model details, see the open-source repository: https://github.com/claim-berlin/CollateralScore.

ChatGPT was employed partially for debugging and adding explanatory comments to the Python code used in the radiomics processing pipeline. All final decisions and validations were made by the authors.

### Statistical analysis

To compare individual clinical variables between the training/cross-validation and independent test sets, we performed univariate statistical analyses. Continuous variables such as age were compared using the Student’s *t*-test. Categorical variables such as Tan score were evaluated using the χ^2^ exact test. SHAP analysis was performed to depict feature importance using the SHAP Python library. For determining the statistical significance in performance between the models, the DeLong test was performed [27]. *p*-values < 0.05 were considered significant.

## Results

### Patient characteristics

The study included 483 patients: 274 for training, 69 for internal testing, and 140 for external testing. Baseline characteristics (age, sex, National Institutes of Health Stroke Scale‒NIHSS, occlusion side) were balanced across groups (all *p* > 0.05). Time from symptom onset to CTA was slightly shorter in the test set (*p* = 0.0915). Insufficient collaterals (Tan score 0–1) comprised 32.9% of patients in the training set, 33.3% in the internal test set, and 29.3% in the external test set, with no significant differences between groups. Detailed patient characteristics are provided in Table 1. A comparison of the internal training set and external test set is provided in Table 2.

**Table 1:** Patient characteristics of the internal train set and the internal test set

Category	Internal train set (*n* = 274)	Internal test set (*n* = 69)	*p*-value
Age (mean ± standard deviation)	64.5 ± 14.0	63.1 ± 13.0	0.457
Female (%)	43.8%	44.9%	0.973
NIHSS (mean ± standard deviation)	17.5 ± 5.6	17.6 ± 5.1	0.877
Onset to CTA (mean ± standard deviation)	155.2 ± 80.2	138.5 ± 70.2	0.092
Right hemisphere symptoms (%)	48.2%	44.93%	0.7279
Middle cerebral artery M1/M2 occlusion (%)	73.4%	73.91%	1.0000
Internal carotid artery occlusion (%)	25.6%	26.09%	1.0000
Insufficient collaterals (Tan score = 0 or 1)	32.9%	33.33%	1.0000

*CTA* Computed tomography angiography, *NIHSS* National Institutes of Health Stroke Scale

**Table 2:** Patient characteristics of the internal training set and the external test set

Category	Internal train set (*n* = 274)	External test set (*n* = 140)	*p*-value
Age (mean ± standard deviation)	64.5 ± 14.0	72.9 ± 15.3	< 0.001
NIHSS (mean ± standard deviation)	17.5 ± 5.6	15.3 ± 7.5	0.002
Right hemisphere symptoms (%)	48.2%	50.7%	0.700
Middle cerebral artery M1/M2 occlusion (%)	73.4%	95.0%	< 0.001
Internal carotid artery occlusion (%)	25.6%	7.2%	< 0.001
Insufficient collaterals (Tan score = 0 or 1)	32.9%	29.3%	0.532

*NIHSS* National Institutes of Health Stroke Scale

### Binary vessels segmentations

To determine the optimal input for radiomics selection, we tested three feature sets on the internal test set. First, using texture features alone, the model achieved an AUROC of 0.8440 (95% confidence interval (CI): 0.736‒0.930) with 89 selected features. Second, combining texture and shape features resulted in an AUROC of 0.778 (95% CI: 0.657‒0.889) with 74 features. Third, using shape features alone (model A), the model achieved the highest internal AUROC of 0.881 (95% CI: 0.770‒0.980) with only 6 selected features (Supplementary Fig. S6) (43 true positives, 16 true negatives, 3 false negatives, 7 false positives). On the external test set, this shape-based model reached an AUROC of 0.8339 (95% CI: 0.707‒0.909) with 85 true positives, 26 true negatives, 14 false negatives, and 15 false positives.

### MCA masks

For the MCA mask model (model B), the radiomics pipeline selected 97 predictive features (Supplementary Fig. S7). On the internal test set, the model achieved an AUROC of 0.8157 (95% CI: 0.707–0.909) with 45 true positives, 10 true negatives, 1 false negative, and 13 false positives 13. On the external test set, the AUROC was 0.663 (95% CI: 0.554–0.765) with 74 true positives, 20 true negatives, 25 false negatives, and 21 false positives.

### Combination of binary and CoW vessel segmentations

The model C was trained using the shape features of the binary segmentations (best-performing radiomics subgroup) and the shape features of the multilabel CoW segmentation. Our selection pipeline resulted in 32 best-performing radiomic features (Supplementary Fig. S8). Although model C performed on the internal test set slightly lower compared to the model using only the shape features from the vessel trees (AUROC 0.8611, 95% CI: 0.748–0.949), it performed better on the external test set, achieving an AUROC of 0.8677 (95% CI: 0.798–0.925). Performance metrics are summarized in Table 3 and Supplementary Figs. S4, S9.

**Table 3:** Comparison of performance metrics between vessel-based and MCA-based models on internal and external test sets

	AUROC (95% CI)	Sensitivity (95% CI)	Specificity (95% CI)	PPV (95% CI)	NPV (95% CI)
Model A, internal test set	0.881 (0.770–0.975)	0.935 (0.848–1.000)	0.696 (0.500–0.900)	0.860 (0.765–0.958)	0.842 (0.650–1.000)
Model B, internal test set	0.816 (0.707–0.909)	0.978 (0.925–1.000)	0.435 (0.238–0.625)	0.776 (0.660–0.873)	0.909 (0.692–1.000)
Model C, internal test set	0.861 (0.748–0.949)	0.9130 (0.833–1.000)	0.6522 (0.476–0.840)	0.8400 (0.736–0.935)	0.7895 (0.600–1.000)
Model A, external test set	0.834 (0.707–0.909)	0.859 (0.780–0.921)	0.634 (0.477–0.788)	0.850 (0.781–0.914)	0.650 (0.500–0.794)
Model B, external test set	0.663 (0.554–0.765)	0.748 (0.660–0.822)	0.488 (0.333–0.647)	0.779 (0.686–0.864)	0.444 (0.302–0.580)
Model C, external test set	0.868 (0.798–0.925)	0.798 (0.716–0.876)	0.829 (0.703–0.935)	0.919 (0.857–0.968)	0.630 (0.508–0.755)

The vessel model significantly outperformed the MCA-based model on both the internal (*p* = 0.003) and external (*p* < 0.001) test sets. Adding the circle of Willis features further improved performance over the vessel-only model (internal test set, *p* = 0.031; external test set, *p* = 0.006)

*AUROC* Area under the receiver operating characteristic curve, *CI* Confidence interval, *MCA* Middle cerebral artery

## Discussion

We present a novel cerebrovascular radiomics approach designed to automate, objectify and improve the assessment of collateral status in patients with acute ischemic stroke. To the best of our knowledge, this is the first approach to leverage radiomic features extracted from vessel segmentations for collateral scoring on CTA. Unlike recent studies that rely on advanced imaging such as multiphase CTA [32] or magnetic resonance perfusion imaging [33], our pipeline utilizes CTA, thus allowing a larger clinical implementation. By providing objective and reproducible scoring, it offers a potential solution to the inter-rater variability associated with manual collateral assessment [34, 35] and, therefore, could increase efficiency and decrease delays, especially in high-volume stroke centers.

Beyond general applicability, several acute clinical scenarios could particularly benefit from automated collateral scoring. First, in borderline cases, such as patients with intermediate Alberta Stroke Program Early CT Score (ASPECTS) [36] or inconclusive perfusion–core mismatch [37], objective collateral quantification could support eligibility decisions for endovascular treatment. Second, in extended time windows, as highlighted by recent trials such as TENSION and RESCUE-Japan LIMIT, reliable assessment of collaterals can identify patients with low or moderate ASPECTS who may still derive benefit from thrombectomy beyond the conventional 6-h window [38, 39]. Finally, automated collateral assessment could streamline workflow by providing rapid and reproducible results, reducing rater-dependent variability, and assisting triage decisions when perfusion imaging is unavailable. Collectively, these aspects underline the translational potential of the proposed method to support evidence-based and time-critical decision-making in acute stroke care. As the assessment would be automated and highly reliable, it could be used to provide more adequate treatment, especially in borderline and late window cases, where the identification of good collaterals can extend the indications for endovascular thrombectomy [6, 38, 39].

Unlike previous radiomics studies on CTA, we evaluated our approach on multicenter internal and external datasets. Cerebrovascular radiomics outperformed traditional MCA atlas-based methods [9] likely due to the broader vascular context captured by vessel segmentation, including anterior and posterior circulations—key regions for collateral recruitment via leptomeningeal pathways. Incorporating CoW features further enhanced performance, likely utilizing information from anatomical variations in the communicating arteries. Based on the SHAP analysis (Fig. S8), the best-performing model identified VoxelVolume derived from the communicating and posterior arteries. This finding aligns with previous reports on the clinical relevance of CoW configurations and thrombus location [40]. Subsequently, the model utilized shape features from both hemispheres to determine collateral status, reflecting decision-making patterns used by neuroradiologists [15, 24].

Beyond acute clinical use, our method can also improve the quality of retrospective datasets. Many stroke outcome prediction models include collateral status as a key factor [41], but inter-rater variability, especially across multicenter data, can reduce their predictive accuracy. In cases where collateral status is unavailable, but imaging data are present, our approach could be used to impute collateral scores. By providing an objective and reproducible collateral assessment, it could serve as a standardized rater, thereby improving the consistency and potentially predictive performance of existing stroke outcome models.

Beyond collateral assessment, our vessel segmentation models, as used in our pipeline, support broader neurovascular applications. Precise vascular quantification may enhance large vessel occlusion detection, addressing a known limitation of currently available tools [42]. Additionally, analyzing vessel morphology (*e.g*., diameter [43], tortuosity [44]) could guide device selection and optimize endovascular thrombectomy strategies for improved recanalization.

This study is limited to proximal LVOs, as they represent the most common and clinically relevant stroke subtype [45]. While collateral assessment may also play a role in the management of distal occlusions [5], collateral scoring in these territories is often less reliable and prone to tilting toward better scores [46]. In the posterior circulation and for multiple vessel occlusions, no widely accepted collateral grading system comparable to the Tan scale currently exists, and perfusion imaging remains the primary tool guiding endovascular thrombectomy decision-making [47]. Therefore, before implementing a similar automated approach for collateral assessment in this region, further studies are required to establish consensus on appropriate grading criteria and to clarify the clinical impact of collateral evaluation.

Model performance depends on segmentation quality. We report the DC and a95HD to include both overlap-based and distance-based evaluation for quantitative evaluation. While segmentation performance could be improved, it remains adequate [48, 49], especially when combined with expert review [50]. Although no major errors were found, minor inaccuracies, especially when distinguishing between intermediate collateral grades (*e.g*., 1 *versus* 2), may affect results (Supplementary Fig. S5a, b—false negative, insufficient, and false positive, sufficient, comparison).

Additionally, the binary left/right division of the vascular tree ignores anatomical variation, potentially misassigning vessels across hemispheres and reducing feature precision (Supplementary Fig. S5a—basilar artery, false positive segmentation). Our preprocessing includes template registration to standardize midline orientation and ensure systematic side attribution, which reduces such errors and preserves the validity of hemispheric comparisons but does not eliminate them. This approach reflects current clinical practice, as the Tan collateral score is based on side-by-side hemispheric comparison. Nevertheless, future developments toward a model capable of fully and reliably segmenting the entire cerebrovascular tree could potentially further enhance model precision and better capture individual vascular variability.

This study represents an initial step toward developing and validating automated collateral status assessment via cerebrovascular radiomics. To assess its clinical utility, future prospective studies with standardized CTA protocols are needed, as variability in contrast timing, artifacts, noise, and reconstruction algorithms may affect vessel classification and collateral prediction (see Supplementary Fig. S5a—false positive and S5b—false negative). Furthermore, scanner-dependent variability, including manufacturer, model, and software differences, should be evaluated alongside radiomics robustness analyses to ensure feature stability and model generalizability. Lastly, clear workflow integration with step-by-step guidance for the use of the tool by the clinicians involved, along with clear instructions for interpreting its output in clinical decision-making, will be essential for successful adoption.

In the next stage, after consensus on the technical aspects, the tool should be evaluated in prospective studies aimed at assessing its clinical utility. Such studies should focus not only on assessment accuracy, but also on time efficiency and treatment inclusivity or exclusivity. For example, how much faster clinical decision-making becomes (*e.g*., door-to-puncture time), how often patients who might previously have been excluded from endovascular therapy are now appropriately included, and the clinical impact of these changes as reflected by functional outcomes such as the 90-day modified Rankin Scale. These evaluations will define the practical and translational value of automated collateral assessment.

In conclusion, this study shows that combining vessel segmentation with radiomics enables objective and accurate collateral assessment. By focusing on vascular structures and automating scoring, our approach offers a generalizable alternative to current grading methods and supports personalized stroke management.

## Supplementary information

**Additional file 1:** **Fig. S1**. Examples of computed tomography angiography exams excluded: (**a**, **b**) Cropped, excluding big vascular territories. (**c**) Motion artifacts. (**d**) Maximum intensity projection reconstruction, voxel size > 2.0 mm. **Fig. S2**. (**a**) Original image. (**b**) Brain mask generation. (**c**) Brain mask-guided cropping. Image after template registration: sagittal view(**d**); axial view (**e**); and coronal view (**f**). **Fig. S3**. Radiomics selection pipeline. (**1**) Extraction of radiomics based on the region of interest. (**2a**) Calculated correlation between the radiomics and create highly correlated pairs (*r* > 0.9). (**2b**) Use of a stratified 10-fold Ridge regression (RIDGE) to calculate feature importance based on the respective task. (**3**) Use of the importance coefficient to exclude from the pairs the less predictive radiomic. (**4**) The top predictive radiomics features are incrementally added to optimize validation through area under the receiver operating characteristic curve (AUROC). (**5**) Random forest classifier training based on radiomics with the best validation AUROC. **Fig. S4**. (**a**) Confusion matrix model A, vessels, internal test set (IT). (**b**) Confusion matrix model A, vessels, external test set (ET). (**c**) Confusion matrix model B, MCA, IT. (**d**) Confusion matrix model B, MCA, ET. (**e**) Confusion matrix Model C, vessels + CoW, IT. (**f**) Confusion matrix model C, vessels + CoW, ET. *CoW* Circle of Willis, *MCA* Middle cerebral artery. **Fig. S5**. Examples of result from model C (vessels + circle of Willis): from the internal test set (**a**); from the external test set (**b**). *FN* False negative, *FP* False positive, *TN* True negative, *TP* True positive. **Fig. S6**. SHAP (Shapley Additive Explanations) analysis of most predictive features: binary vessels model. **Fig. S7**. SHAP (Shapley Additive Explanations) analysis of most predictive features: middle cerebral artery masks. **Fig. S8**. SHAP (Shapley Additive Explanations) analysis of most predictive features: Binary and Circle of Willis. Compare with Fig. S6. **Fig. S9**. Receiver operating characteristic (ROC) curve analysis for the internal test set (**a**) and the external test set (**b**) for all models.

## Data Availability

The full Python-based pipeline is available at https://github.com/claim-berlin/CollateralScore. Clinical data and imaging are not publicly available.

## Abbreviations

ASPECTS: Alberta Stroke Program Early CT Score
AUROC: Area under the receiver operating characteristic curve
CoW: Circle of Willis
CTA: Computed tomography angiography
LVO: Large vessel occlusion
MCA: Middle cerebral artery
ROI: Region of interest
SHAP: SHapley Additive exPlanations
