# Supplementary material for: Cerebrovascular CTA radiomics for objective collateral grading in acute ischemic stroke
Source: Eur Radiol Exp. 2026 Mar 16;10:27. doi: 10.1186/s41747-026-00680-8 (PMC12992882; doi:10.1186/s41747-026-00680-8)
Supplement: Supplementary file 1 — Additional file 1: Fig. S1. Examples of computed tomography angiography exams excluded: (a, b) Cropped, excluding big vascular territories. (c) Motion artifacts. (d) Maximum intensity projection reconstruction, voxel size > 2.0 mm. Fig. S2. (a) Original image. (b) Brain mask generation. (c) Brain mask-guided cropping. Image after template registration: sagittal view(d); axial view (e); and coronal view (f). Fig. S3. Radiomics selection pipeline. (1) Extraction of radiomics based on the region of interest. (2a) Calculated correlation between the radiomics and create highly correlated pairs (r > 0.9). (2b) Use of a stratified 10-fold Ridge regression (RIDGE) to calculate feature importance based on the respective task. (3) Use of the importance coefficient to exclude from the pairs the less predictive radiomic. (4) The top predictive radiomics features are incrementally added to optimize validation through area under the receiver operating characteristic curve (AUROC). (5) Random forest classifier training based on radiomics with the best validation AUROC. Fig. S4. (a) Confusion matrix model A, vessels, internal test set (IT). (b) Confusion matrix model A, vessels, external test set (ET). (c) Confusion matrix model B, MCA, IT. (d) Confusion matrix model B, MCA, ET. (e) Confusion matrix Model C, vessels + CoW, IT. (f) Confusion matrix model C, vessels + CoW, ET. CoW Circle of Willis, MCA Middle cerebral artery. Fig. S5. Examples of result from model C (vessels + circle of Willis): from the internal test set (a); from the external test set (b). FN False negative, FP False positive, TN True negative, TP True positive. Fig. S6. SHAP (Shapley Additive Explanations) analysis of most predictive features: binary vessels model. Fig. S7. SHAP (Shapley Additive Explanations) analysis of most predictive features: middle cerebral artery masks. Fig. S8. SHAP (Shapley Additive Explanations) analysis of most predictive features: Binary and Circle of Willis. Compare with Fig. S6. Fig. [file 41747_2026_680_MOESM1_ESM.pdf]

Cerebrovascular CTA radiomics for objective collateral grading in acute ischemic stroke.

ELECTRONIC SUPPLEMENTARY MATERIAL

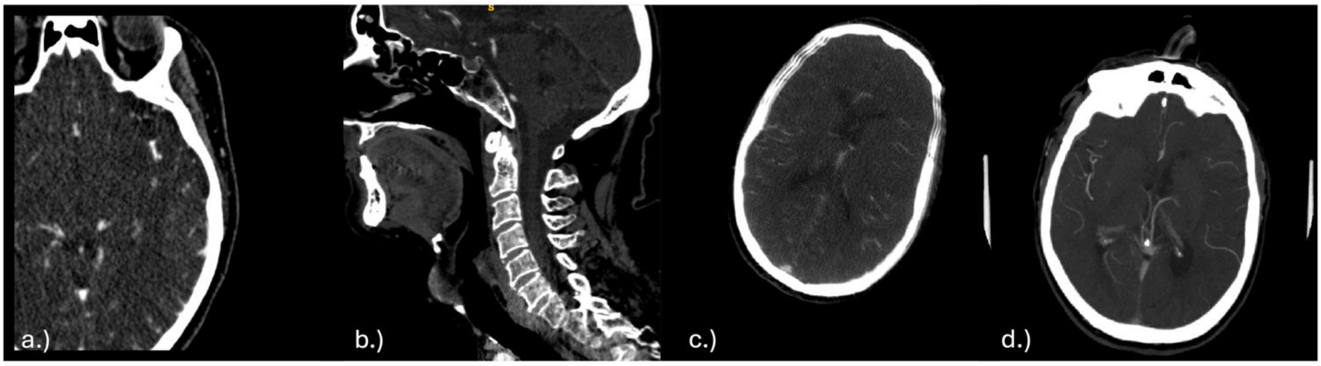

Fig. S1 Examples of computed tomography angiography exams excluded: (**a**, **b**) Cropped, excluding big vascular territories. (**c**) Motion artifacts. (**d**) Maximum intensity projection reconstruction, voxel size > 2.0 mm.

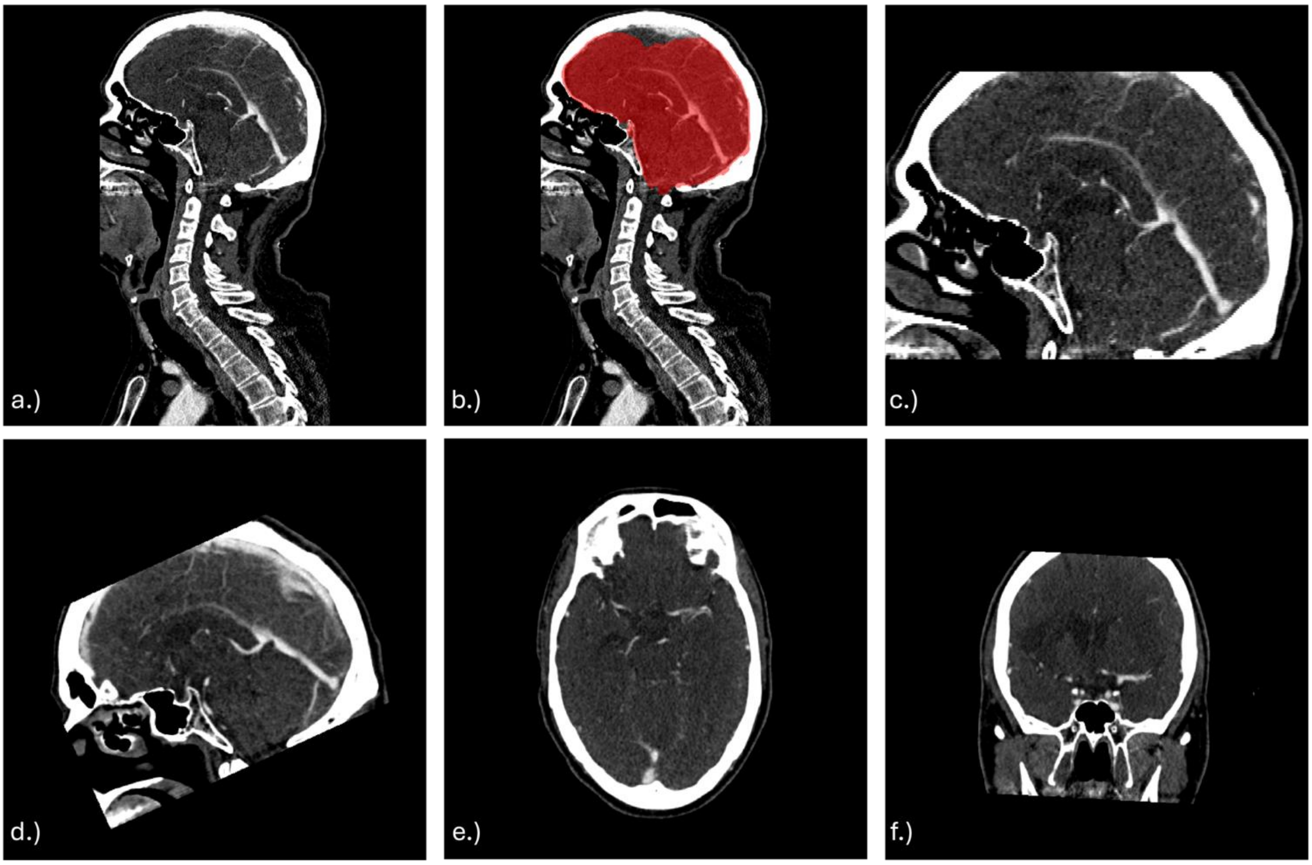

Fig. S2 (**a**) Original image. (**b**) Brain mask generation. (**c**) Brain mask-guided cropping. Image after template registration: sagittal view(**d**); axial view (**e**); and coronal view (**f**).

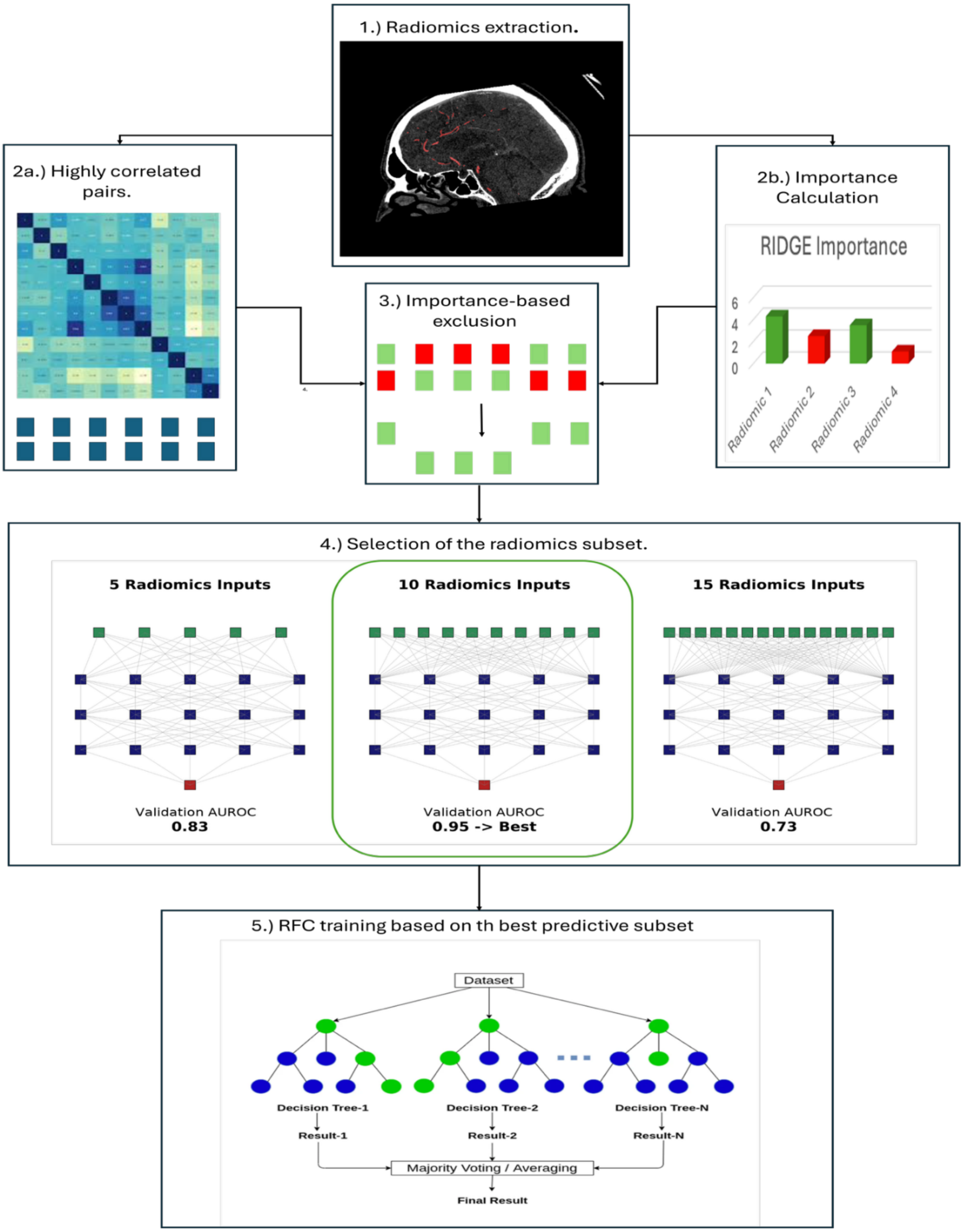

Fig. S3 Radiomics selection pipeline. (**1**) Extraction of radiomics based on the region of interest. (**2a**) Calculated correlation between the radiomics and create highly correlated pairs ($r > 0.9$). (**2b**) Use of a stratified 10-fold Ridge regression (RIDGE) to calculate feature importance based on the respective task. (**3**) Use of the importance coefficient to exclude from the pairs the less predictive radiomic. (**4**) The top predictive radiomics features are incrementally added to optimize validation through area under the receiver operating characteristic curve (AUROC). (**5**) Random forest classifier training based on radiomics with the best validation AUROC.

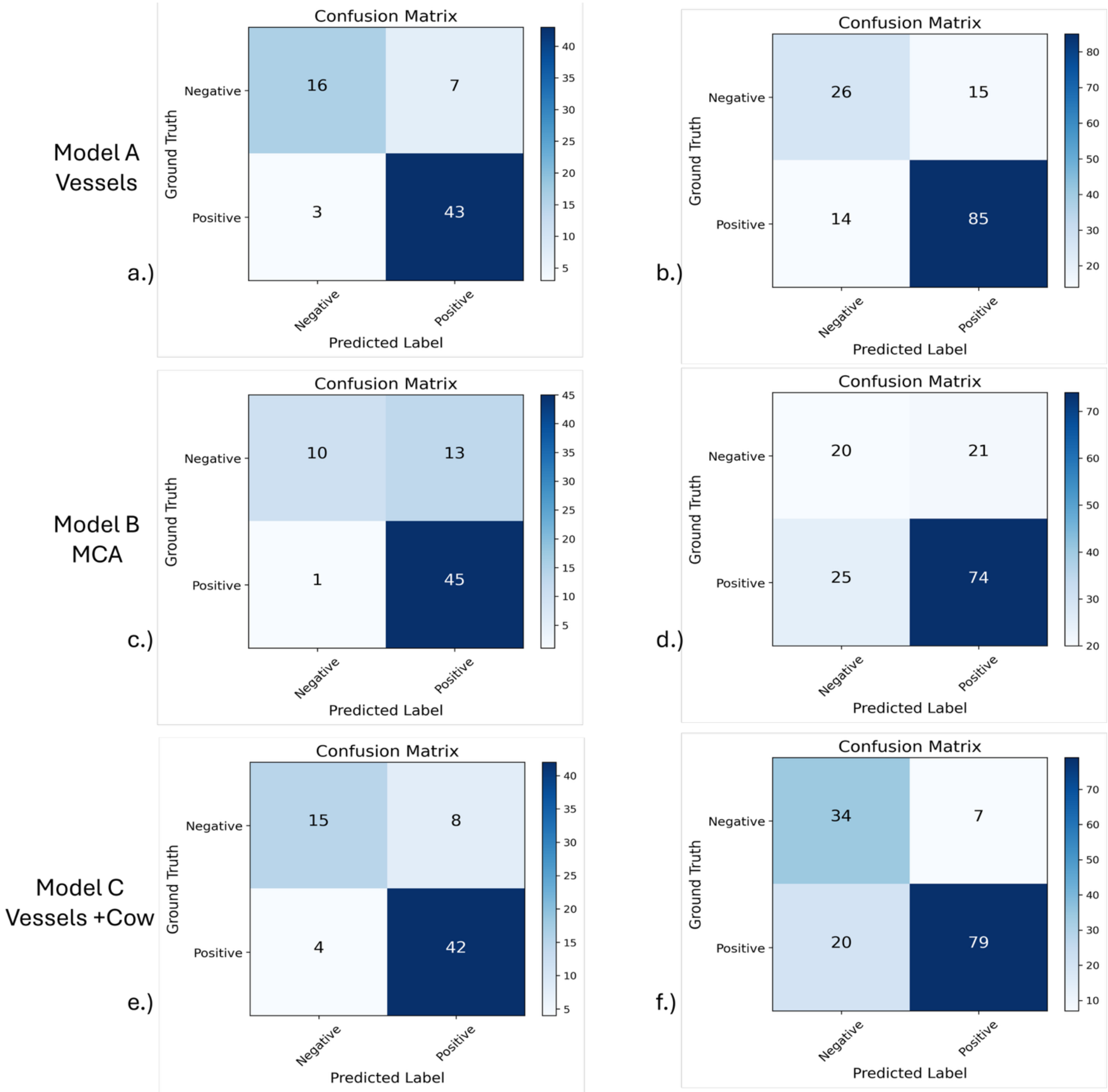

Fig. S4 (**a**) Confusion matrix model A, vessels, internal test set (IT). (**b**) Confusion matrix model A, vessels, external test set (ET). (**c**) Confusion matrix model B, MCA, IT. (**d**) Confusion matrix model B, MCA, ET. (**e**) Confusion matrix Model C, vessels + CoW, IT. (**f**) Confusion matrix model C, vessels + CoW, ET. *CoW* Circle of Willis, *MCA* Middle cerebral artery.

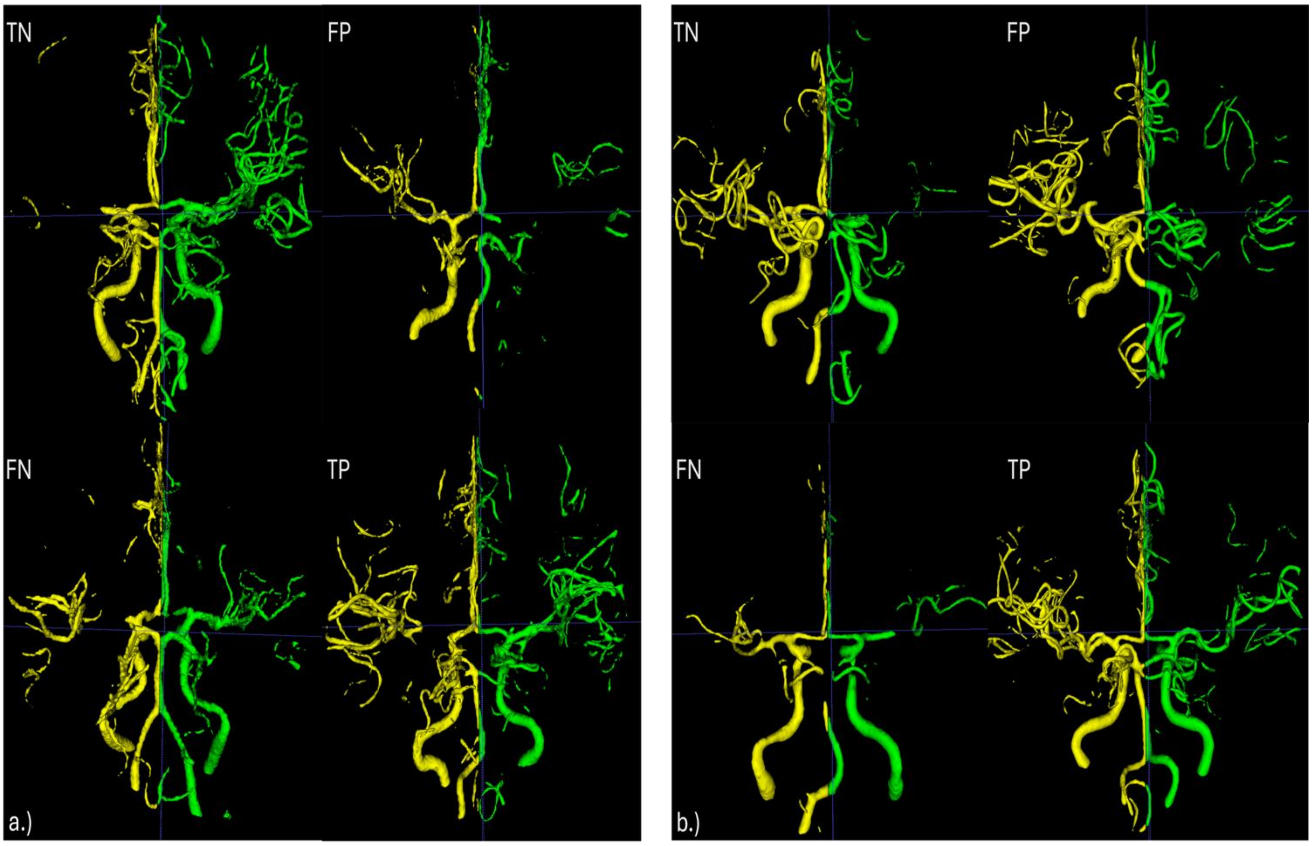

Fig. S5 Examples of result from model C (vessels + circle of Willis): from the internal test set (**a**); from the external test set (**b**). *FN* False negative, *FP* False positive, *TN* True negative, *TP* True positive.

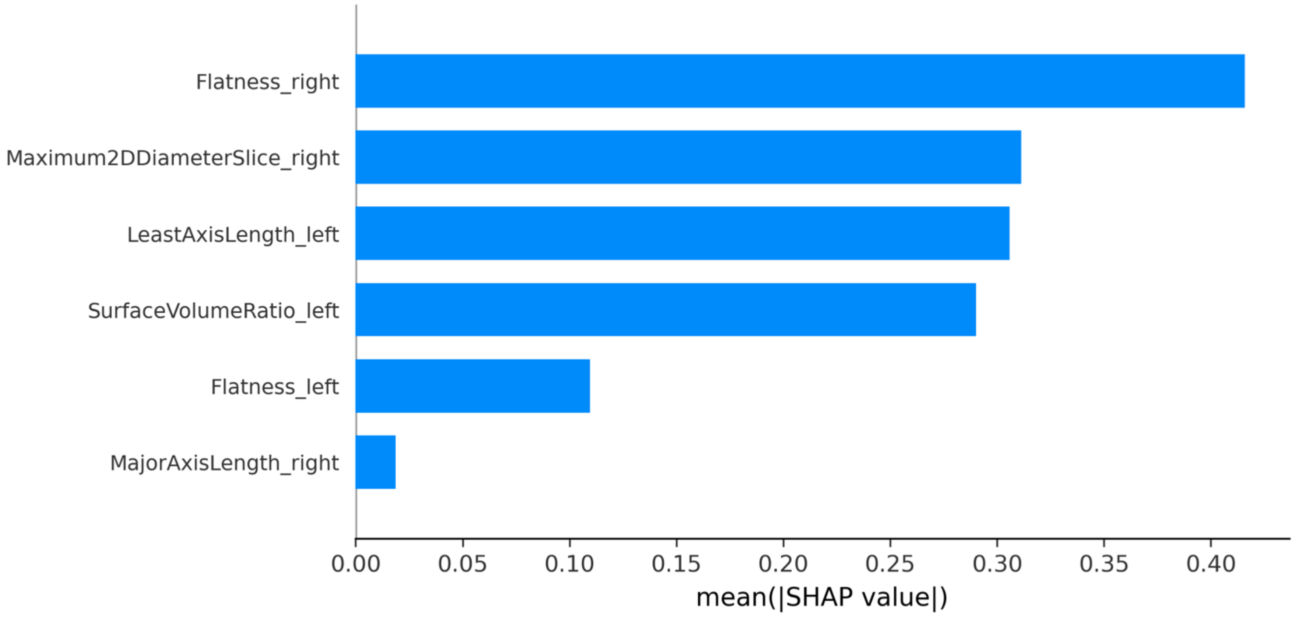

Fig. S6 SHAP (Shapley Additive Explanations) analysis of most predictive features: binary vessels model.

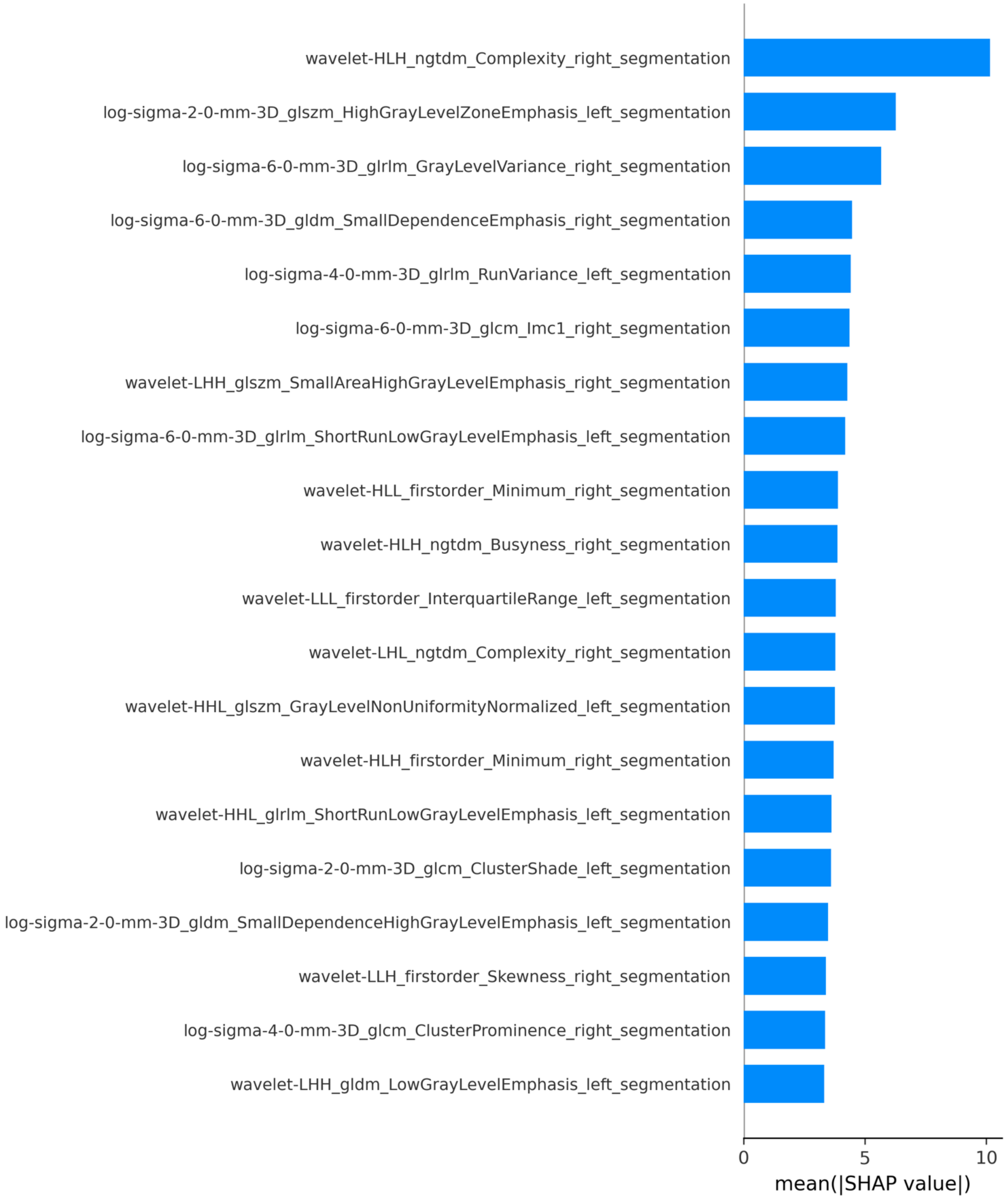

Fig. S7 SHAP (Shapley Additive Explanations) analysis of most predictive features: middle cerebral artery masks.

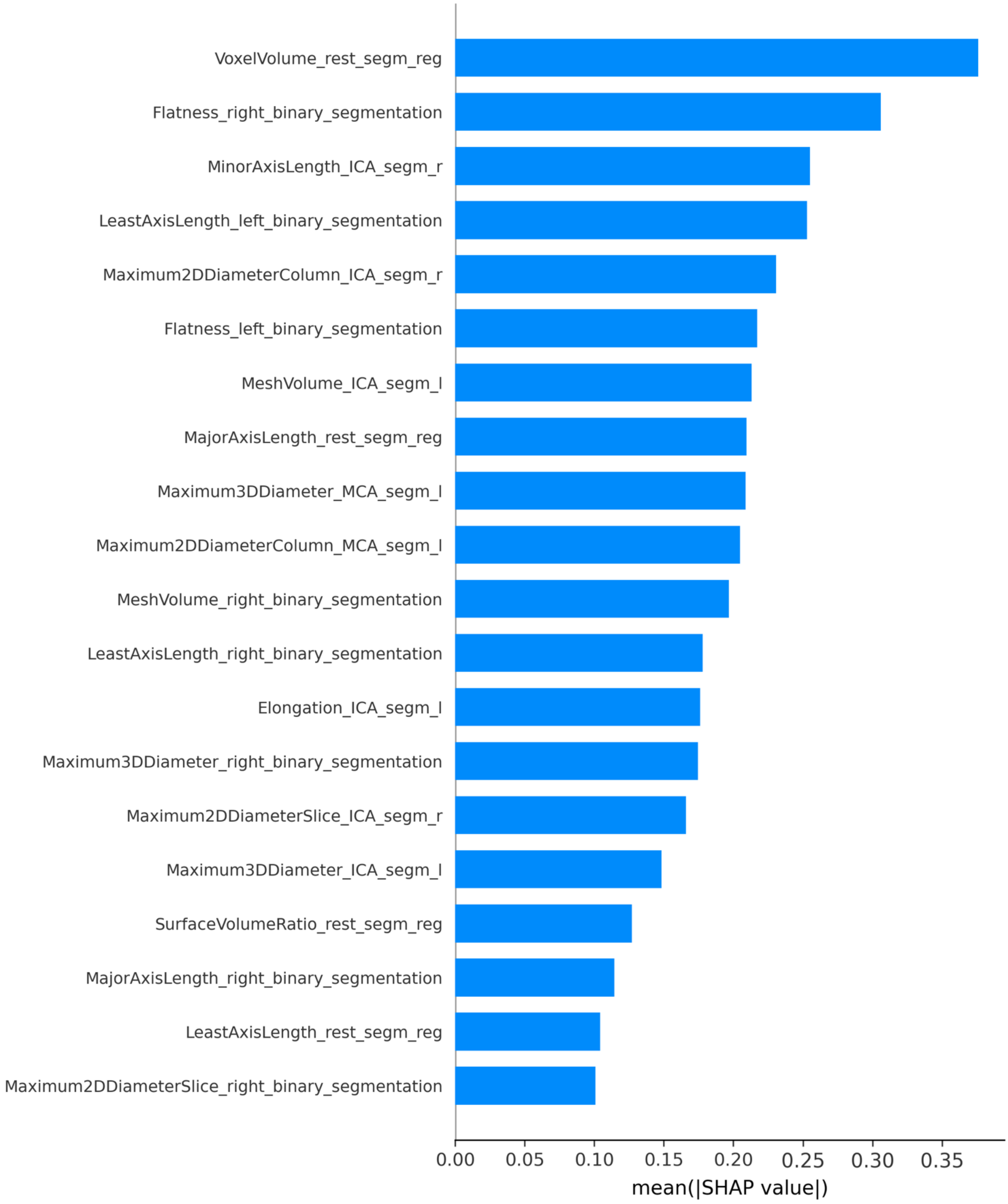

Fig. S8 SHAP (Shapley Additive Explanations) analysis of most predictive features: Binary and Circle of Willis. Compare with Fig. S6.

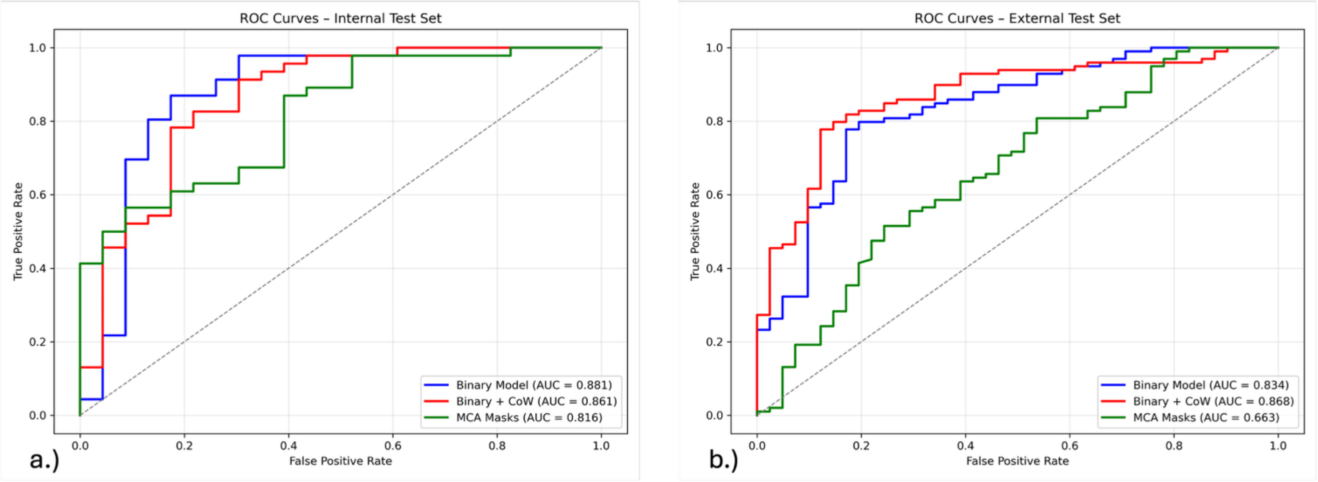

Fig. S9 Receiver operating characteristic (ROC) curve analysis for the internal test set (**a**) and the external test set (**b**) for all models.
